# Predictors of Independent Community Ambulation in Individuals with Chronic Stroke: A Cross-Sectional Study of Gait Speed, Gait Endurance, and Balance Self-Efficacy

**DOI:** 10.3390/jcm14248649

**Published:** 2025-12-06

**Authors:** SeungHeon An, DongGeon Lee, DongMin Park, Kyeongbong Lee

**Affiliations:** 1 Department of Gait Lab, National Rehabilitation Center, Seoul 01022, Republic of Korea; 2 Department of Physical Therapy, Sinsegae Rehabilitation Hospital, Changwon 51216, Republic of Korea; 3 Department of Physical Therapy, Zenith Rehabilitation Hospital, Seoul 04993, Republic of Korea; 4 Department of Physical Therapy, Kangwon National University, Samcheok 25949, Republic of Korea

**Keywords:** walking, walking speed, physical endurance, self-efficacy, stroke

## Abstract

**Background/Objectives**: Community ambulation after stroke depends on locomotor capacity and confidence in everyday environments. We compared functional performance across three community walking levels and identified constructs independently associated with being an independent community walker in individuals with chronic stroke. **Methods**: Adults admitted to an acute-care general hospital or an inpatient rehabilitation hospital were enrolled. Community walking level was classified by a self-reported questionnaire. Primary constructs were gait speed, gait endurance, and balance self-efficacy measured with standard clinical tests. Additional measures described balance, lower-limb motor function, and task-based mobility. Group differences were examined with one-way analysis of variance with Bonferroni comparisons. Community walking status was modeled with binary logistic regression using forward stepwise selection. **Results**: Fifty-nine individuals were analyzed. Performance differed across levels. Effect sizes were small, medium, or large overall. Independent community walkers showed faster gait speed, longer walking distance, and higher balance self-efficacy, with the same direction for balance and lower-limb motor scores and shorter times on task-based tests. In univariable models, age, sex, and time since stroke were not associated with independence, whereas assistive device use related to lower odds. In the multivariable model, gait speed, gait endurance, and balance self-efficacy retained independent associations with independent community walking. Other measures were not retained after adjustment. **Conclusions**: Community walking status in chronic stroke relates most closely to gait speed, gait endurance, and balance self-efficacy. Evaluation can emphasize the 10 m Walk Test, 6 Min Walk Test, and Activities-specific Balance Confidence Scale, with impairment and task-based tests used to guide intervention planning.

## 1. Introduction

Restoring gait function is a primary goal of post-stroke rehabilitation [1], and many individuals with stroke eventually regain independent gait [2]. Nevertheless, only a smaller proportion accomplishes independent community ambulation within the early months after stroke, despite its importance for daily functioning and social participation [3]. Community ambulation is described as walking in complex everyday environments, including public venues and habitual places, rather than simple walking in controlled settings [4]. Accordingly, community ambulation has developed as a critical factor in enhancing mobility and facilitating social participation among individuals with stroke.

Assessment of community ambulation in rehabilitation practice and research primarily relies on gait speed and endurance [5,6]. Even at discharge, 32% to 47% of individuals with a Functional Ambulation Category (FAC) score of ≥4 remain unable to ambulate independently in the community, indicating that conventional discharge status does not ensure community-level mobility [3]. A previous study reported no significant differences in gait speed among several lower FAC groups [7]; however, discriminant analysis still identified gait speed as a significant predictor of ambulation classification, with a prediction accuracy of 44% [8]. Gait speed estimates differ across studies because protocols vary in walkway length and in the handling of acceleration and deceleration [9], contributing to comfortable gait speed thresholds for community ambulation that range from 0.78 to 1.2 m/s [10,11].

Gait endurance is integral to community ambulation, and the 6 Min Walk Test (6MWT) is an established measure of functional capacity in stroke with demonstrated safety and feasibility in acute care [12]. Distance-based thresholds suggest that individuals may require approximately 288 to 500 m to manage community ambulation demands [13], and step-count-based classifications have been investigated to reflect daily activities [11]. However, it may not adequately estimate environmental adaptability, dual-task demands, or psychosocial influences that shape community ambulation in various circumstances [14]. Collectively, these limitations support a multidimensional approach that integrates physical capacity measures, such as gait speed and gait endurance, with psychological constructs, including balance self-efficacy, when evaluating community ambulation after stroke.

Measures of balance self-efficacy in stroke demonstrate good reliability and construct validity [15], higher balance self-efficacy is associated with better physical function and perceived health status after stroke [16]. In chronic stroke, balance self-efficacy relates to activity and participation, indicating relevance to everyday mobility [17]. Balance self-efficacy contributes independently to community reintegration after stroke, which supports including self-efficacy alongside performance-based assessments when studying community ambulation [18].

Therefore, the present study aimed to compare functional performance across community ambulation levels and to identify constructs independently associated with independent community ambulation in individuals with chronic stroke. We prespecified gait speed, gait endurance, and balance self-efficacy as primary constructs. We hypothesized that higher gait speed, greater gait endurance, and higher balance self-efficacy would each demonstrate independent associations with independent community ambulation after adjustment for relevant demographic and clinical variables. The objective was to define a concise set of clinically actionable indicators to guide assessment and goal setting for community ambulation in physical therapy.

## 2. Materials and Methods

### 2.1. Study Design

This was a cross-sectional study examining functional performance and balance self-efficacy associated with community ambulation in individuals with chronic stroke. Participants were recruited from two inpatient rehabilitation hospitals, an acute-care general hospital in Changwon and a stroke rehabilitation hospital in Seoul, Republic of Korea. The study protocol was approved by the Institutional Review Board of Kangwon National University (KWNUIRB-2025-04-007-001), and all participants provided written informed consent.

### 2.2. Participants

Individuals with chronic stroke were recruited from two inpatient rehabilitation hospitals: an acute-care general hospital in Changwon, and a stroke rehabilitation hospital in Seoul. Eligible patients had a clinical diagnosis of post-stroke hemiparesis at least 6 months prior and were receiving multidisciplinary rehabilitation that included medical care, nursing, physical therapy, and occupational therapy. This study was approved by the Institutional Review Board of Kangwon National University (KWNUIRB-2025-04-007-001). All participants provided written informed consent after receiving a full explanation of the study procedures.

Inclusion criteria were: (1) Mini-Mental State Examination–Korean version (MMSE-K) score ≥ 24; (2) ability to walk independently for at least 10 m with or without an assistive device; (3) absence of lower-limb orthopedic conditions that could affect gait. Exclusion criteria were: (1) contraindications to the 6MWT according to established guidance, including unstable angina, recent myocardial infarction, uncontrolled cardiovascular disease, resting heart rate ≥ 120 beats per minute, systolic blood pressure ≥ 180 mmHg, or diastolic blood pressure ≥ 110 mmHg [19]; (2) current use of medications that could compromise balance; (3) recurrent stroke or bilateral hemiparesis.

The required sample size was estimated a priori using G*Power 3.1.9.7 for binary logistic regression. A moderate effect size of 0.30 was chosen based on a previous study that investigated community ambulation after chronic stroke using comparable functional predictors and multivariable modeling [20]. With an effect size of 0.30, α = 0.05, power = 0.95, and 14 predictors, the minimum required sample size was 56 participants.

### 2.3. Procedure

After written informed consent was obtained, baseline characteristics were collected by chart review and a standardized patient questionnaire administered by a physical therapist. Collected variables included age, time since stroke, diagnosis, side of hemiparesis, MMSE-K score, and use of a walking assistive device.

A trained assessor administered a self-reported questionnaire developed to classify community ambulation ability after stroke [21]. The questionnaire asks patients to choose the single option that best reflects their real-life walking outside the home without any prompting toward specific answers. Response options were: (1) no difficulty walking in the community and no need for physical assistance or supervision; (2) slight difficulty and need for supervision when walking longer distances away from home; (3) moderate difficulty and need for supervision when walking near home and at longer distances; (4) severe difficulty and consistent need for another person’s physical assistance; (5) does not walk outside the home. For analysis, participants were classified as independent community walkers [response (1)], limited community walkers [responses (2)~(4)], or household walkers [response (5)].

Functional assessments were performed over two to three days in random order. All assessments were administered by a physical therapist with more than 20 years of experience in neurological rehabilitation. A trained assistant stood beside the participant during testing to ensure safety and prevent falls without interfering with the procedures. To minimize fatigue, a rest period of 2 to 5 min was provided between assessments. Participants were permitted to use their customized assistive device during all tests if needed. All procedures were completed under standardized instructions and testing conditions, and data were confirmed immediately after each session to confirm completeness before proceeding to subsequent assessments.

The following outcome measures were obtained. The Functional Ambulation Category (FAC) was used to rate the level of assistance required for ambulation. Comfortable walking speed was assessed with the 10 m Walk Test (10mWT). Walking endurance was assessed with the 6MWT on a rectangular track designed to include turning distances. Additional measures included the ABC Scale, Berg Balance Scale (BBS), Fugl-Meyer Assessment of the Lower Extremity (FMA-L/E), Five Times Sit-to-Stand Test (5xSTS), Timed Up and Go Test (TUG), Figure-of-8 Walk Test (F8WT), and Four Square Step Test (FSST).

### 2.4. Measurements

The FAC was used to rate gait ability based on the amount of human assistance required during ambulation, irrespective of assistive device use. The FAC is a six-point ordinal scale from 0 to 5, and higher scores indicate greater independence. Ratings indicate the need for physical assistance or supervision during walking rather than the presence of a cane or walker. Participants were assessed while walking in a level indoor corridor with their usual footwear and devices, and the highest level that safely described performance was recorded. Inter-rater reliability in individuals with stroke has been reported as excellent (κ = 0.950) [22].

Comfortable gait speed was assessed with the 10mWT. Participants walked along a 14 m walkway that included 2 m acceleration and 2 m deceleration zones, which were excluded from measurement. The time required to traverse the central 10 m, from when the leading foot crossed the 2 m mark until it passed the 12 m mark, was recorded in seconds. Three trials were performed, and the mean value was used for analysis. Test–retest reliability has been reported with an intraclass correlation coefficient (ICC) of 0.95 [23].

Gait endurance and functional capacity were evaluated with the 6MWT. This test quantifies the total distance walked during six minutes at a self-selected pace under standardized instructions, and has excellent test–retest reliability in stroke, with a pooled ICC of 0.98, indicating stable performance across repeated assessments [24].

Balance self-efficacy and concern about falling during daily and community activities were assessed with the ABC Scale [25]. Participants rated their confidence for sixteen everyday tasks such as walking on uneven surfaces, crossing a street, using stairs, and reaching while standing on a scale from 0 to 100, and the overall score was calculated as the mean percentage across all items, with higher values indicating greater self-efficacy. Test–retest reliability in chronic stroke has been reported as good, with an ICC of 0.85 [15].

Balance performance was evaluated with the BBS across sitting, standing, and postural transitions [26]. The scale includes fourteen items scored from 0 to 4 for a maximum of 56 points, and higher totals indicate better balance. Administration followed standardized instructions using common equipment such as an armless chair, stopwatch, ruler, and a step or stool, and tasks cover sit-to-stand, standing unsupported, turning, reaching, and stepping, which together reflect static and dynamic balance. Inter-rater reliability in stroke has been reported as excellent, with ICC ranging from 0.92 to 0.98 [27].

Lower-limb motor impairment was assessed with the FMA-L/E [28]. The assessment comprises seventeen items scored from 0 to 2, yielding a total of thirty-four points, and higher totals indicate less impairment. The scoring reflected selective control, reflex activity, and coordination at the hip, knee, and ankle. High intra-rater and inter-rater reliability in stroke has been reported [29].

Lower-limb strength and transitional movement were assessed with the 5xSTS [30]. Participants rose from a chair with a backrest and no armrests five times as quickly as possible, with arms crossed over the chest and without upper-limb support. Timing started on the verbal cue to begin and stopped when the participant fully returned to sitting at the end of the fifth repetition. Inter-rater reliability has been reported as excellent with an ICC of 0.99 [31].

Functional mobility was assessed with the TUG, the F8WT, and the FSST. For the TUG, the time required to rise from a standard armchair, walk 3 m, turn, return, and sit down was measured [32]. The mean of three trials was used as the final score. Test–retest reliability has been reported as excellent, with an ICC of 0.96 [33].

For the F8WT, participants walked a figure-of-8 pathway defined by two cones placed 1.52 m apart, and an additional 0.61 m of space was provided at each end [34]. Starting at the midpoint, they circled the left cone counterclockwise, returned to center, and then circled the right cone clockwise. Total time from the first step until return to the starting point was recorded. Inter-rater reliability has been reported as excellent, with an ICC of 0.99 [35].

For the FSST, participants stepped over four cylindrical rods arranged in a cross pattern and followed a predefined sequence of forward, right, backward, and left, then reversed the sequence to return to the starting square [36]. Time was measured from the first step until the final step back to the starting position. Trials were repeated if a rod was touched, a boundary was crossed, both feet were not placed within a square, or assistance was required [37].

### 2.5. Statistical Analysis

All analyses were performed with SPSS for Windows, version 29.0 (IBM Corp., Armonk, NY, USA). Normality of continuous variables was examined with the Shapiro–Wilk test. Continuous variables are reported as mean ± standard deviation and categorical variables as frequency and percentage.

The three community walking levels were compared for each continuous outcome using one-way analysis of variance. Assumptions were examined with the Shapiro–Wilk test for normality and Levene’s test for homogeneity of variance. When the overall F test was significant, pairwise comparisons were performed with Bonferroni adjustment. If homogeneity of variance was violated, Welch analysis of variance and Games–Howell comparisons were applied. The magnitude of between-group differences was quantified with eta-squared, with 0.01 interpreted as small, 0.06 as medium, and 0.14 as large [38].

The associations of personal factors and functional measures with community ambulation status were examined using binary logistic regression. The dependent variable was coded as independent community walkers equal to 1 and non-community walkers equal to 0. The non-community category combined limited community walkers and household walkers. Odds ratios with 95 percent confidence intervals were reported.

The variables that reached statistical significance in the univariable models were entered into a multivariable binary logistic regression using forward stepwise selection based on the Wald statistic. The final model identified factors independently associated with independent community ambulation after accounting for other significant predictors. Statistical significance was set at *p* < 0.05.

## 3. Results

Allowing for a 20% attrition rate, 67 patients were enrolled. After withdrawals (*n* = 2), incomplete assessments (*n* = 3), emergent discharge (*n* = 2), and other reasons (*n* = 1), data from 59 individuals were included in the analyses. They were predominantly male, the mean age was approximately 60 years, and the average time since stroke was about two years. Ischemic stroke was more frequent than hemorrhagic stroke, right-sided hemiparesis was slightly more common, and cognitive status was generally preserved. Summary values for clinical and functional measures are shown in Table 1.

Community ambulation level yielded three groups, independent community walkers, limited community walkers, and household walkers. Performance differed across groups. The independent group demonstrated faster gait speed, longer walking distance on gait endurance testing, and higher balance self-efficacy. They also showed higher scores on balance and lower-limb strength and shorter times on task performance tests. These patterns are consistent with the one-way analyses and post hoc comparisons (Table 2).

As shown in Table 3, univariable logistic models indicated that personal factors, including age, sex, and time since stroke, were not associated with independent community ambulation. Use of a walking aid was related to lower odds of independence. Several functional measures were significant correlates of independent walking status, including the ambulation category, gait speed, endurance, balance self-efficacy, balance performance, lower-limb motor score, and times on transitional and curved or multidirectional walking tasks.

In the multivariable model using forward stepwise selection, three variables retained independent associations with independent community ambulation, faster gait speed, greater walking endurance, and higher balance self-efficacy. Other measures did not remain significant after adjustment for these constructs (Table 4).

## 4. Discussion

This cross-sectional study compared functional performance across three community walking levels in individuals with chronic stroke and identified constructs associated with independent community ambulation. In multivariable analyses, greater gait speed on the 10mWT, longer walking distance on the 6MWT, and higher balance self-efficacy on the ABC Scale showed independent associations with community walking status. The overall pattern indicates that community mobility after stroke depends on locomotor capacity and on perceived capability to handle balance challenges encountered in daily environments. The present findings do not allow conclusions regarding the effectiveness of specific physiotherapy techniques.

As a clinical proposal informed by the constructs retained in the multivariable model, future intervention studies may consider combined training that aims gait speed, lower limb resistance, and balance self-efficacy. This could include speed focused gait practice, progressive resistive exercise to support propulsion and postural stability, and structured strategies to strengthen balance self-efficacy such as graded exposure to community walking tasks and feedback on task command. These elements are presented as directions for rehabilitation training and should be tested in longitudinal trials.

Across the three groups of independent community walkers, limited community walkers, and household walkers, functional performance showed a consistent pattern that ordered the groups by mobility level. The independent group demonstrated faster gait speed on the 10mWT, longer walking distance on the 6MWT, and higher balance self-efficacy on the ABC Scale. The same direction of differences was observed for BBS and FMA-L/E, and tests that emphasize turning and multidirectional stepping such as the TUG, F8WT, and FSST also separated the groups with shorter completion times in those who achieved independent community walking.

These findings agree with a previous study that classifications based on gait speed and endurance are closely related to community mobility after stroke and that balance self-efficacy contributes to performance outside the home [39]. Evidence also supports dynamic balance tests including the FSST as useful for distinguishing mobility status and fall risk, and the F8WT differentiates curved-path walking demands that commonly arise in everyday environments [40].

From a clinical perspective, the ordered pattern across groups supports an assessment approach that focus on gait speed with the 10mWT, gait endurance with the 6MWT, and balance self-efficacy with the ABC Scale to characterize community walking capacity. The BBS and FMA-L/E can describe balance and lower-limb motor impairment, and the TUG, F8WT, and FSST can indicate task-specific practice that targets transitions, turning, curved-path walking, and multidirectional stepping. This structure maintains efficiency for decision making in rehabilitation and aligns testing with the community walking demands reflected in the present results.

Univariable logistic regression showed that age, sex, and time since stroke were not associated with independent community walking, whereas use of a walking aid was related to lower odds of independence. Functional measures reflecting independent walking, gait speed, walking endurance, balance self-efficacy, balance performance, lower-limb motor function, and task-based mobility were each associated with community walking status. Similar results have been reported, with gait speed and endurance frequently linked to community walking status in stroke cohorts and balance self-efficacy emerging as a correlate of activity and participation [41]. Dynamic balance measures are also supported, with FSST demonstrating validity and reliability in ambulant individuals after stroke, and F8WT differentiating curved-path walking demands that reflect everyday environments [42].

These convergent univariable findings support a measurement strategy that gives priority to gait speed by 10mWT, gait endurance by 6MWT, and balance self-efficacy by ABC Scale when identifying individuals who are likely to achieve independent community walking. Balance and motor scales can define impairment severity, and time-based mobility tests can guide task-specific progression during therapy. This approach aligns the evaluation focus with constructs that showed meaningful associations in the present data and with contemporary reports on community mobility after stroke [39].

In the multivariable logistic regression, gait speed on the 10mWT, walking endurance on the 6MWT, and balance self-efficacy on the ABC Scale showed unique associations with independent community walking after adjustment [43]. These measures represent short-distance velocity, sustained ambulatory capacity, and perceived capability to manage balance challenges during daily walking, and together provide a concise assessment of key constructs required for community mobility.

Balance self-efficacy, as assessed by the ABC Scale, likely reflects multidimensional influences beyond perceived balance alone. Confidence during community walking may be developed by fear of falling, anxiety related to environmental demands, prior walking experiences, and motivational factors, which are not fully separable from physical capacity. Accordingly, balance self-efficacy may function not only as a retained construct in the multivariable model but also as a potential mediator or moderator in the association between locomotor capacity and independent community mobility. Because this interpretation remains conceptual and mediation or moderation was not assessed in the present cross-sectional analysis, future longitudinal studies should apply mediation or moderation modelling to clarify how physical capacity and psychological factors jointly influence independent community mobility after stroke.

Although other balance and motor measures did not remain statistically significant in the final multivariable model, this should not be understood as a lack of clinical relevance. Several of these tests were significant in univariable models and distinguished community walking levels, but they likely share variance with gait speed, endurance, and self-efficacy [44]. Thus, once these primary constructs were included, the additional measures provided limited incremental information within this model. Clinically, BBS, TUG, FSST, and lower-limb motor scores remain useful for describing impairment severity and task-specific mobility demands.

Clinically, these findings support a concise assessment strategy that emphasizes the 10mWT, 6MWT, and ABC Scale for screening, stratification, and goal setting for community walking. Impairment measures such as the BBS and FMA-L/E, along with task-based tests including the TUG, F8WT, FSST, and 5xSTS, remain useful for describing deficits and identifying task-specific mobility limitations that inform intervention planning. Rehabilitation programs may combine speed- and endurance-oriented gait practice with structured approaches to strengthen balance self-efficacy, consistent with evidence that interventions targeting balance self-efficacy can produce measurable benefits [45].

This study examined performance-based measures associated with community ambulation, yet community walking reflects the interaction of physical capacity, psychological factors, and adaptation to everyday environments, and several limitations should be noted. First, the cross-sectional design cannot establish temporal order or causality between community ambulation and the measured variables. Second, psychological constructs such as motivation, anxiety, depressive symptoms, satisfaction, stress, and fatigue were not assessed, and environmental features relevant to walking were not included, so residual confounding is possible. Third, the sample size was modest, which constrained the number of predictors in multivariable models and may have reduced precision; participants were relatively young with mild to moderate impairment, and recruitment from two hospitals within one region limits generalizability. In addition, the multivariable model was developed using forward selection in a modest sample. Variable selection procedures in limited samples may increase the risk of overfitting and lead to unstable coefficient estimates. Thus, the retained predictors should be considered as investigative associations that require confirmation in independent datasets. Fourth, community ambulation status was determined solely by a self-reported questionnaire. Self-report may overestimate gait performance in daily life and does not adequately reflect day-to-day variability in community walking exposure, environmental demands, or contextual constraints. Consequently, misclassification of true community mobility is possible, and objective validation was not available in the present study. Future research should proceed in a coherent sequence. To begin with, confirmation of these associations is needed in larger, multicenter cohorts using prespecified predictor sets and modern regression approaches that reduce overfitting, with internal validation such as bootstrap resampling or cross-validation, and balancing or cautiously validated synthetic augmentation when group imbalance persists. Similar strategies have been implemented in gait- analysis datasets for rare neurological conditions, where combinations of class balancing, generative synthetic augmentation, stratified cross-validation, and Bayesian optimization improved classification performance in small, imbalanced samples [46]. Next, studies should include psychological and environmental measures with core performance tests such as the 10mWT, 6MWT, and ABC Scale. In addition, they should incorporate wearable or location-based monitoring of community mobility. Such monitoring could provide objective indices of gait parameters and patterns across daily life, and the environmental conditions encountered during community walking, thereby reducing reliance on self-report and improving measurement transparency. Because many of these devices are low burden and feasible in routine community settings, objective mobility metrics derived from wearable sensors are increasingly accessible and are likely to refine community ambulation classification in future stroke cohorts. Finally, they should also validate classification thresholds across settings to clarify how capacity and context influence independent community ambulation after stroke.

## 5. Conclusions

Independent community ambulation in individuals with chronic stroke was most closely related to gait speed, gait endurance, and balance self-efficacy. In multivariable analyses, the 10mWT, 6MWT, and ABC Scale each showed independent associations with independent community walking, while other measures did not remain significant after adjustment. These findings support an evaluation plan that prioritizes the 10mWT, 6MWT, and ABC for screening and goal setting, with additional tests used to describe balance and motor impairment when needed. Clinical programs that combine speed-focused and endurance-focused gait practice with strategies to strengthen balance self-efficacy may help improve community mobility, although confirmatory longitudinal studies are required.

## Figures and Tables

**Table 1:** Clinical characteristics and outcome measures of the subjects (*n* = 59).

Parameters	Values
Age (year)	60.54 ± 13.98 (28–76)
Sex (male/female)	37 (62.7)/22 (37.3)
Onset (months)	27.83 ± 24.08 (7–110)
Diagnosis (infarction/hemorrhage)	39 (66.1)/20 (33.9)
Paretic side (left/right)	26 (44.1)/33 (55.9)
MMSE-K (score)	26.03 ± 1.74 (24–30)
Use of walking aid None/Single point cane/Quadri-pod cane	28 (47.5)/15 (25.4)/16 (27.1)
Functional Ambulation Category (score)	4.20 ± 0.92 (2–5)
10 m Walk Test (m/s)	0.73 ± 0.30 (0.18–2.13)
6 Min Walk Test (m)	259.18 ± 102.44 (82.10–674.63)
ABC Scale (score)	68.32 ± 26.27 (12–98)
Berg Balance Scale (score)	41.31 ± 11.96 (12–56)
FMA–L/E (score)	23.19 ± 6.80 (8–34)
5xSTS (s)	20.62 ± 12.13 (8.35–68.43)
Timed Up and Go (s)	18.17 ± 10.39 (8.10–65.23)
Figure-8 Walk Test (s)	20.91 ± 15.05 (6.83–80.99)
Four Square Step Test (s)	28.60 ± 21.59 (5.50–130.01)

Note: Values are presented as n (%) or mean ± standard deviation, with the range reported as minimum to maximum. SD indicates standard deviation. Abbreviation: MMSE-K, Mini Mental Status Evaluation—Korean; ABC Scale, Activities-specific Balance Confidence Scale; FMA–L/E, Fugl-Meyer Assessment of the Lower Extremity.

**Table 2:** Comparison of functional performance assessments among participants (*n* = 59).

Outcome Measures	Independent Community Walkers (A) (*n* = 26)	Limited Community Walkers (B) (*n* = 17)	Household Walkers (C) (*n* = 16)	F	*p*	Post Hoc
FAC (score)	4.54 ± 0.76	4.18 ± 0.81	3.69 ± 1.08	4.751	0.012 *	A ∣ C
10mWT (m/s)	0.96 ± 0.25	0.67 ± 0.18	0.42 ± 0.13	34.509	0.001 ***	A ∣ B ∣ C
6MWT (m)	341.36 ± 82.36	231.43 ± 56.06	155.10 ± 42.26	41.095	0.001 ***	A ∣ B ∣ C
ABC scale (score)	85.31 ± 14.33	66.41 ± 27.05	42.75 ± 18.55	22.938	0.001 ***	A ∣ B ∣ C
BBS (score)	48.08 ± 8.98	40.65 ± 12.73	34.25 ± 11.96	5.659	0.006 **	A ∣ C
FMA–L/E (score)	26.31 ± 5.32	21.59 ± 7.88	19.81 ± 5.81	6.072	0.004 **	A ∣ C
5xSTS (s)	16.77 ± 8.36	19.96 ± 10.73	27.57 ± 12.13	4.421	0.012 *	A ∣ C
TUG (s)	12.38 ± 3.18	19.37 ± 10.24	26.33 ± 12.58	12.75	0.001 ***	A ∣ B C
F8WT (s)	13.31 ± 4.47	19.56 ± 8.15	34.0 ± 21.61	14.937	0.001 ***	A B ∣ C
FSST (s)	16.07 ± 4.60	28.95 ± 13.01	48.62 ± 29.77	17.779	0.001 ***	A B ∣ C

Note: Values are presented mean ± standard deviation. * *p* < 0.05. ** *p* < 0.01, *** *p* < 0.001. Abbreviations: FAC, Functional Ambulation Category; 10mWT, 10 m Walk Test; 6MWT, 6 Min Walk Test; ABC scale, Activities-specific Balance Confidence Scale; BBS, Berg Balance Scale; FMA-L/E, Fugl-Meyer assessment-lower/extremity; 5xSTS, Five Times Sit-to-Stand Test; TUG, Timed Up and Go Test; F8WT, Figure-of-8 Walk Test; FSST, Four Square Step Test.

**Table 3:** Binary logistic regression analysis of the relationship between community ambulation and personal and functional performance.

Variables	Regression Coefficient	SE	*p*-Value	EXP (B)
Age	−0.21	0.019	0.281	0.979
Gender	0.354	0.558	0.525	1.425
Duration	−0.014	0.012	0.231	0.986
Use of walking aid	−0.668	0.267	0.012 *	0.513
FAC (score)	0.894	0.349	0.01 **	2.444
10mWT (m/s)	3.905	4.213	0.001 ***	2.702
6MWT (m)	0.041	0.011	0.001 ***	1.042
ABC scale (score)	0.068	0.018	0.001 ***	1.071
BBS (score)	0.076	0.028	0.007 **	1.079
FMA–L/E (score)	0.148	0.049	0.003 **	1.160
5xSTS (s)	−0.061	0.030	0.041 *	0.941
TUG (s)	−0.265	0.077	0.001 ***	0.767
F8WT (s)	−0.213	0.060	0.001 ***	0.808
FSST (s)	−0.249	0.079	0.002 **	0.779

Note: Dependent variable: Independent community walkers (1), Non-community walkers (0) = Limited community walkers & Household walkers. * *p* < 0.05. ** *p* < 0.01, *** *p* < 0.001. Abbreviations: SE: Standard error; EXP (B), odds ratio; FAC, Functional ambulation category; 10mWT, 10 m Walk Test; 6MWT, 6 Min Walk Test; ABC scale, Activities-specific Balance Confidence Scale; BBS, Berg Balance Scale; FMA-L/E, Fugl-Meyer assessment-lower/extremity; 5xSTS, Five Times Sit-to-Stand Test; TUG, Timed Up and Go Test; F8WT, Figure-of-8 Walk Test; FSST, Four Square Step Test.

**Table 4:** Multivariate logistic regression for community ambulation.

Variables	B	SE	Wald	*p*-Value	EXP (B)
Use of walking aid	1.607	1.257	1.634	0.201	4.986
FAC (score)	2.370	2.605	0.828	0.363	10.695
10mWT (m/s)	0.113	0.053	4.510	0.034 *	1.119
6MWT (m)	0.060	0.026	5.440	0.020 *	1.061
ABC scale (score)	0.110	0.054	4.177	0.041 *	1.116
BBS (score)	−0.402	0.233	2.970	0.085	0.669
FMA–L/E (score)	−0.216	0.180	1.433	0.234	0.806
5xSTS (s)	0.053	0.151	0.126	0.723	1.055
TUG (s)	0.315	0.410	0.592	0.442	1.370
F8WT (s)	0.088	0.349	0.064	0.800	1.092
FSST (s)	−0.654	0.364	3.233	0.072	0.520
Constant	−6.935	14.255	0.237	0.627	0.001

Note: Dependent variable: Independent community walkers (1), non-community walkers (0) = Dependent community walkers & Household walkers. * *p* < 0.05. Abbreviations: B, Regression coefficient; SE, Standard error; EXP (B), odds ratio; FAC, Functional ambulation category; 10mWT, 10 m Walk Test; 6MWT, 6 Min Walk Test; ABC scale, Activities-specific Balance Confidence Scale; BBS, Berg Balance Scale; FMA-L/E, Fugl-Meyer assessment-lower/extremity; 5xSTS, Five Times Sit-to-Stand Test; TUG, Timed Up and Go Test; F8WT, Figure-of-8 Walk Test; FSST, Four Square Step Test.

## Data Availability

The data presented in this study are available on request from the corresponding author.

## Abbreviations

The following abbreviations are used in this manuscript:

FAC	Functional Ambulation Category
6MWT	6 Min Walk Test
MMSE-K	Mini-Mental State Examination–Korean version
10mWT	10 m Walk Test
ABC Scale	Activities-specific Balance Confidence Scale
BBS	Berg Balance Scale
FMA-L/E	Fugl-Meyer Assessment–Lower Extremity
5xSTS	Five Times Sit-to-Stand Test
TUG	Timed Up and Go Test
F8WT	Figure-of-8 Walk Test
FSST	Four Square Step Test
